# Silica Materials for Medical Applications

**DOI:** 10.2174/1874120700802010001

**Published:** 2008-01-29

**Authors:** María Vallet-Regí, Francisco Balas

**Affiliations:** Department of Inorganic and Bioinorganic Chemistry, Facultad de Farmacia, Universidad Complutense de Madrid (UCM) and Networking Biomedical Engineering Research Center for Bioengineering, Biomaterials and Nanomedicine (CIBER-BBN), Plz. Ramón y Cajal, s/n., 28040 Madrid, Spain

## Abstract

The two main applications of silica-based materials in medicine and biotechnology, *i.e.* for bone-repairing devices and for drug delivery systems, are presented and discussed. The influence of the structure and chemical composition in the final characteristics and properties of every silica-based material is also shown as a function of the both applications presented. The adequate combination of the synthesis techniques, template systems and additives leads to the development of materials that merge the bioactive behavior with the drug carrier ability. These systems could be excellent candidates as materials for the development of devices for tissue engineering.

## 1. INTRODUCTION

The ceramics for medical practice have been traditionally classified into two main groups, those inert materials such as alumina, zirconia or carbon [1,2] and those that undergo a specific interaction with the physiological environment when are implanted that leads to the material integration in the living tissue. These latter materials are known as bioactive ceramics and include some calcium phosphates,[3]. glasses and glass-ceramics [4]. In the latter area of research in the biomedical field, the silica-based ceramics receive a great interest. The first materials to be considered bioactive were certain compositions of silica-based glasses, leading to the development of a wide area of research on new silica materials for biomedical applications. It can be assumed bioactivity as the ability that show certain materials, with a definite chemical composition and structure, when are submitted to the physiological environment, which consists in a series of chemical processes in the material-living tissue interface that leads to the material incorporation into the living tissue. Such processes are verified through the nucleation and growth on the material surface of a layer of carbonated hydroxyapatite with low-crystallinity and close-related to the mineral phase of bone tissues.

This process was first described by Hench *et al.* in the 1970s [5] in some compositions of silica glasses. Since then many studies, comprising different glasses and calcium silicate compositions, have shown *in vitro *bioactivity [6,7]. Although the mechanism of apatite formation has not been completely elucidated, the presence of silanol groups in the surface seems to be crucial. In this sense, several authors propose that silanol groups act as nucleation sites. However, there are more factors compulsory for apatite formation [8]; among them, it is interesting to point out the textural properties, i.e. those related with the porosity. It has been proved that there is a direct relation between both pore size and volume and the nucleation rate of the apatite layer [9].

Further insights in the research on silica-based materials are the bioactive organic-inorganic hybrids, which show also interesting properties for clinical applications [10-15]. Hard tissues in vertebrates are natural composite materials, therefore the synthesis of such composites seems to be a quite reasonable approach to imitate Nature in the laboratory. However, the scope of the silica materials with clinical applications has considerably changed in the latest years. For instance, it is worth mentioning the research effort carried out in mesoporous materials for designing biomedical devices with two main uses, drug delivery systems and bone tissue regeneration [16,17]. This double perspective of the mesoporous materials, the proper combination of the bioactive behavior of silica with the open texture and ordered porosities, has propelled the research in this area in the latest years. Moreover, the so-called *templated glasses *have been obtained using surfactants as structure directing agents [18].

Summarizing, the two main landmarks in the use of silica-based ceramics in biomedicine are the development of materials useful for tissue regeneration and in drug delivery systems. Both research areas are strongly related, as it can be observed in the current literature and nowadays bioactive materials can be designed with the drug release properties. The fact is the relationship between different areas has lead to the opening of the research scope on silica materials for biomedical applications. In this sense, in order to help in the making of the present review article and to facilitate the reading and understanding, the scheme in Fig. (**1**) is included. In that, the diverse applications in biomedicine of the different silica-based materials will be presented and discussed.

## 2. AMORPHOUS SILICA SPHERES

Monodispersed silica spheres having a homogeneous diameter in the sub-micrometer range can be used as a starting material for building devices having a wide range of technological applications [19-21]. From a different perspective, sub-micrometer sized silica spheres can be surface functionalized with different organic ligands and, by doing so, the field is opened to practical applications in chromatography, selective separations and biological immunoassays [22], to mention only a few examples. On the other hand, silica microbeads can easily be incorporated into micro fluidic devices, thus enhancing the sensitivity for detection of trace amounts of biological molecules.

**Fig. (2):**
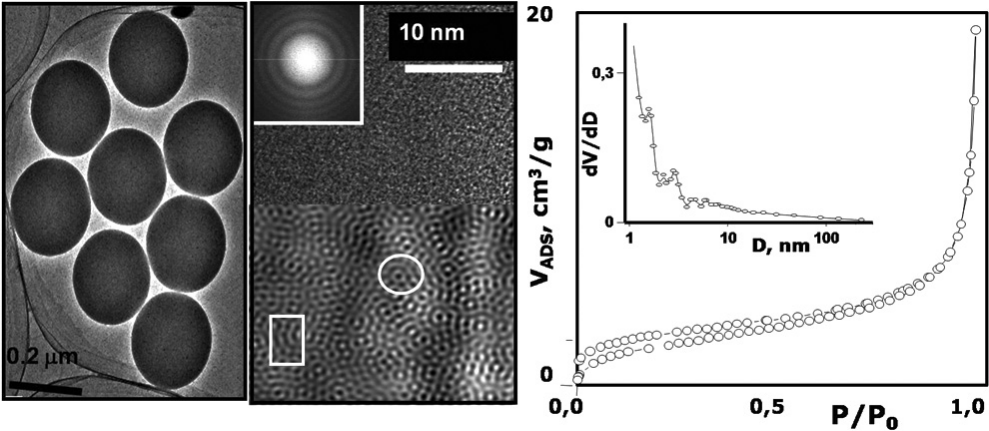
TEM image of SiO_2_ microspheres obtained by the Stöber method. The HR-TEM image shows the microstructure of SiO_2_.

The simplest way to synthesize silica with spherical shape is the Stöber method [23]. Using this procedure spheres can be synthesized in a large variety of sizes by controlling the pH conditions in the synthetic environment. Fig. (**2**) shows that Stöber silica microspheres show very homogeneous particle sizes and at microstructure level are amorphous. This can be assured by the broad and diffuse electron scattering rings observed in the electron diffraction (ED) pattern. The Fourier-transform filtered image (Fig. **2**) shows that several local crystalline and quasi-crystalline blocks can detected in the silica spheres, which are in agreement with the distribution of SiO_2_ clusters in silica-based amorphous materials used for biomedical applications [13,16]. Moreover, the pore volume and diameter of the silica microspheres is usually lower that that found for silica in bulk state, which is usually attributed to the fractal dimension of the surface of spheres. In such case, the surface is held in the outermost surface of materials, as it has been shown by using small angle X-ray scattering studies [24].

The surface of silica spheres that can be synthesized using sol-gel techniques is highly reactive and therefore can be modified by functionalization with the adequate organic reagents. This latter process is generally used for producing an active layer for the adsorption of molecules for drug delivery applications. For these purposes, the silica spheres serve as vectors for transporting the desired drug molecule through the vascular system to the desired target organ [25,26].

### 2.1 Nanospheres of SiO_2_-γFe_2_O_3_

Silica spheres in the nanometric range easily can be directed through the human body if the spherical particles show any characteristic that can be externally controlled. In this sense, the development of magnetic nanoparticles is also focusing the attention of many researchers because of their broad range of possible applications in not only in electronics, catalysis, and magnetic memories,[27,28]. but also in biomedicine [29]. In this latter area, the magnetic nanospheres have been used for magnetic bioseparations [30], hyperthermia of tumors [31], contrast agents for magnetic resonance imaging (MRI) [32] and for designing drug carriers [33]. Magnetic nanoparticles as drug delivery vectors provide the ability to selectively target the desired organs or tissues inside the body, as well as accumulate a certain concentration of nanoparticles along the therapy path by means of the application of an external magnetic field.

The process to incorporate magnetic species into the silica spherical particles has to be performed for avoiding the direct contact between the magnetic core and the tissue while keeping the stability of the colloid in the biological environment. The most successful attempts to carry out this kind of silica nanospheres are the aerosol-assisted routes [34,35]. Fig. (**3**) shows the microstructure and magnetic characterization of silica spheres encapsulating maghemite nanoparticles [36]. It can be observed that the magnetization curves of the encapsulated nanoparticles are characteristic of a superparamagnetic material in agreement with the small size of the magnetic cores in the silica spheres. The superparamagnetic behavior is particularly valuable for hyperthermia, where the nanoparticles could generate heat as a result of a combination of Néel and Brownian mechanisms [37].

## 3. BIOGLASSES

According to Zachariasen [38], a glass is a substance which can form extended three dimensional networks lacking periodicity with energy content comparable with that of the corresponding crystal network. In essence, the density and the mechanical properties of glass are solid-like; however, the atoms form a continuous random network such that the unit cell (in crystal structure terminology) is infinitely large, containing an infinite number of atoms. A working definition of glass is that it is a solid with a liquid-like structure [39].

The characteristic amorphicity of these glasses is generally a product of the structure of silicate glass, usually defined by [SiO_4_] tetrahedra. While crystallised silica shows a uniform arrangement of these tetrahedra, as could be expected of any crystalline material, this is not the case in silica glass. Indeed, for the generation of a glass it is needed a network forming agent, for which the tetrahedral silica units is an outstanding candidate. In addition, silica shows enhanced characteristics for developing bioactive materials, as it has been mentioned in the Introduction.

A common feature of both the crystalline -*i.e.*, quartz- and the amorphous structures is that each oxygen ion links two tetrahedra, although forming a more open arrangement in the case of amorphous silica. Such a rather open structure facilitates the inclusion of cations referred to as network modifiers, and this feature allows a wide range of silica glasses to be obtained.

The presence of cations, such as Na^+^, K^+^ and Ca^2+^, in the glass causes a discontinuity of the glassy network through the disruption of some siloxane bridging bonds (Si-O-Si). As a consequence, non-bridging oxygen is released. Traditionally, for the melt glasses, the objective of such network modifiers was there to break a proportion of those bonds, thus allowing the melt to solidify with a high degree of disorder. Its presence provided lower melting temperatures and viscosity values, reducing the economic costs of glass production, while ensuring a high degree of disorder.

Nevertheless, such disordered structure, enhanced by the presence of network modifiers, gives rise to the high reactivity of these glasses in aqueous environments. This high reactivity is the main advantage of their application in periodontal repair and bone augmentation, since it enhances the bioactive behavior in physiological fluids resulting in the crystallization of the apatite-like phase [40-42]. Early bioactive glasses were prepared by the classic quenching of melts comprising SiO_2_ and P_2_O_5_ as network formers and CaO and Na_2_O as network modifiers [5]. In the early 1990s, sol-gel processing was introduced for the synthesis of bioactive glasses [43], allowing higher purity and homogeneity in the compositions as well as longer ranges of textural properties [44].

The bioactive behavior of the sol-gel glasses has been extensively shown since the early 1990s in a wide variety of silica-based compositions [6,45-48], even with modifications in the synthesis procedure [49]. Fig. (**4**) shows the transmission electron microscopy (TEM) and scanning electron microscopy (SEM) images of a sol-gel bioglass composed by 80% SiO_2_, 17% CaO and 3% P_2_O_5_; the so-called _80_Si_17_Ca_3_P composition after immersion in a simulated body fluid (SBF) for 7 days at 37ºC [50]. As it can be seen, the surface is fully covered by a layer of crystalline aggregates. More information on this layer can be drawn from the high-resolution TEM images, where it can be observed that such aggregates are formed by needle-like nanocrystals with sizes about 50 nm. Electron diffraction patterns show that the observed crystalline phase can be identified as apatite similar to the bone mineral phase. Moreover, Fig. (**4**) also shows that the thickness of the deposited apatite layer is thicker than 2 μm and is tightly bonded to the substrate.

Although the bioactivity of glasses is a very interesting property of the materials in order to design devices for prosthetic applications, the sol-gel glasses show poor mechanical properties. As it can be seen in Fig. (**5**), the fracture toughness of sol gel glasses, regardless of the bioactive character, is very low to be considered for repairing large bone defects. In this sense, two alternatives have been proposed to enhance the mechanical properties of the materials with bioactive behavior; the bioactive glass ceramics and the Star-gels.

### 3.1 Bioactive Glass-Ceramics

A glass ceramic is defined as material where one or several crystal phases are embedded in a glassy matrix, which covers at least the 90% of the total volume of the material. In the special case of silica-based glassy matrices, the application of glass ceramics was particularly remarkable in the biomedical world. The earlier reports of Kokubo in the 1980s centered the attention to these materials as potential candidates for the production of prosthetic devices with a good bioactive behavior. The most interesting material within this type was composed of apatite and wollastonite (A-W glass-ceramic), which showed not only bioactivity but also improved mechanical properties compared to those of pure glassy systems. In Fig. (**5**) are depicted the averaged fracture toughness of the glass ceramics. Due to this improvement of the mechanical resistance, glass ceramics have been proposed for prosthetic devices in load-bearing conditions.

Actually, the bioactive properties of glass-ceramics are more difficult to predict than those of glasses, especially *in vivo*. Hench [51] developed three different microstructural 45S5 compositions: glass, partially crystalline and fully crystalline, and after 6 weeks *in vivo* all the implants were bonded to the cortical bone of a rat femur, proving that the presence of a silica phase is enough for a noticeable bioactivity. Moreover, further research shown that the A-W glass ceramic also bonds to living bone by means of a layer rich in calcium phosphates, although with no silica gel layer detected at the implant-bone interface. Apparently, the presence of crystalline phases can be a hindrance to the bioactive behavior of glass-ceramics [52].

However, the research done on bioactive glass ceramics in the latest years have produced several new compositions, in which the good mechanical properties are conserved but the bioactivity has reached values comparable to those of sol-gel glasses [6,53,54]. These novel glass ceramics are obtained by properly merging together the knowledge in the synthesis of sol-gel glasses, which are used as precursors, with heating and annealing procedures common in the preparation of melt glasses. The obtained glass ceramics, therefore, show microstructural characteristics close to those of parent sol-gel glasses that improve the bioactivity and, moreover, the mechanical behavior is equivalent to that of the classical A-W based systems.

### 3.2 Star Gels

In 1995 DuPont Corp. developed a novel series of silica based materials that were called star gels [55]. Star gels are a type of organic–inorganic hybrid with a unique structure of an organic core surrounded by flexible arms, which are terminated in alkoxysilane groups. At the macroscopic level, star gels exhibit an intermediate behavior, in terms of mechanical properties, between conventional glasses and highly cross-linked rubbers. Currently, star gels are still one of the most interesting subjects in the field of hybrid materials [56]. The synthesis of materials able to integrate with bone while preserving the mechanical properties of star gels, would mean a very important advance in materials science for biomedical applications. These materials can be excellent candidates for bone tissue regeneration when star gels are obtained as monoliths of different shapes in order to fit into any kind of medium or large bone defect. In addition, the star gels must be structurally homogeneous so that their biological and mechanical response when implanted can be safely predicted and, finally, star gels must be able to develop an apatite-like phase in contact with physiological fluids, *i.e.* must be bioactive.

Bioactive star gels can be obtained as monoliths of any shape and size and are able to develop an apatite phase on their surface when soaked in simulated body fluid. Morphologically and structurally are homogeneous and are far better than conventional bioactive glasses from a mechanical point of view. Therefore, bioactive star gels could be excellent candidates for osseous regeneration in medium and large bone defects [57]. In addition, as it can be seen in Fig. (**5**), the fracture toughness of the bioactive star-gels are much better than those of sol-gel glasses and close related to the fracture toughness of the cortical bone.

## 4. ORDERED MESOPOROUS SILICA

Ordered mesoporous silica-based materials constitute an amazing family of solids that show ordered arrangements of pores as channels and cavities with different geometries built up from [SiO_4_] tetrahedra. The pore sizes of these materials are always very homogeneous ranging from 2 to 50 nm and can be controlled and modified, in a reasonable extension, using several synthetic strategies [58,59]. The most common an well-known ordered mesoporous frameworks are the 2D hexagonal planar, with symmetry group *p*6*mm*, MCM-41 [58] and SBA-15 [59] structures with pores around 2 and 10 nm, respectively, and the 3D-cubic MCM-48 [58], with symmetry group Ia¯3*d* with pore size about 3 nm.

The most important feature of these materials, from a practical point of view, is the feasibility of synthesize the mesoporous frameworks with various different pore sizes and geometries. This fact opens a wide range of possibilities for hosting molecules larger than the ones exhibited in the traditional microporous host materials; the zeolites and related materials. This is of great interest for designing materials able to be used as drug carriers, since the pore size of these materials are similar in magnitude to the molecular size of drugs. In view of all of these facts, mesoporous materials have been developed as drug delivery systems since 2001 [60]. The importance of these materials as drug carriers is based on the ability of the silanol groups in the mesopore walls to adsorb molecules of pharmacological interest, followed by a potentially controlled release that depends of several factors [61,62]. These pioneer works have opened new directions in the research on drug delivery systems based in ordered mesoporous silica [63-71]. In Fig. (**6**) is depicted an scheme of the kind of drug molecules that can be adsorbed in the pores of ordered mesoporous silicas.

The adsorption capacity of the silica walls between adjacent pores can be modulated through functionalization with different chemical species depending on the molecule to be adsorbed. The chemical modification of the surface of mesoporous materials is a common strategy for modulating the surface properties and the performance in the desired conditions. The literature of mesoporous materials holds a huge amount of functionalization procedures over different inorganic matrices. The main functionalization procedures currently in use are the co-condensation procedure [72], in which the organic functional group is mixed with the silica precursors and all the procedure is carried out in the same reaction vessel; and the post-synthesis grafting method [73], where the already formed inorganic silica matrix is reacted with the functionalizating agent in anhydrous conditions to yield organically modified silica mesopores. Chemically grafting functional groups on the ordered mesoporous network originates a noticeable change in the adsorption characteristics of the silica surface as well as in its polarity. The chemical modification of the silanol groups at the pore walls has to be selected depending on the drug molecule to get the desired loading and release [74].

The influence of the chemical interaction between silica matrix and drug molecule on the adsorption and delivery rate of drugs in functionalized mesoporous materials has been further investigated [75]. The wide range of available modifications helps to select the more suitable organic group depending on the drug to be confined and delivered. This fact evidences the possibility of designing the mesoporous matrix depending on the molecule to be adsorbed and delivered, and also depending on the dosage and release kinetics desired for a given therapy. Therefore, several factors influence in the final adsorption properties of the mesoporous silicas, when intended for developing materials for drug delivery. These factors are schematically shown in Fig. (**7**).

**Fig. (8):**
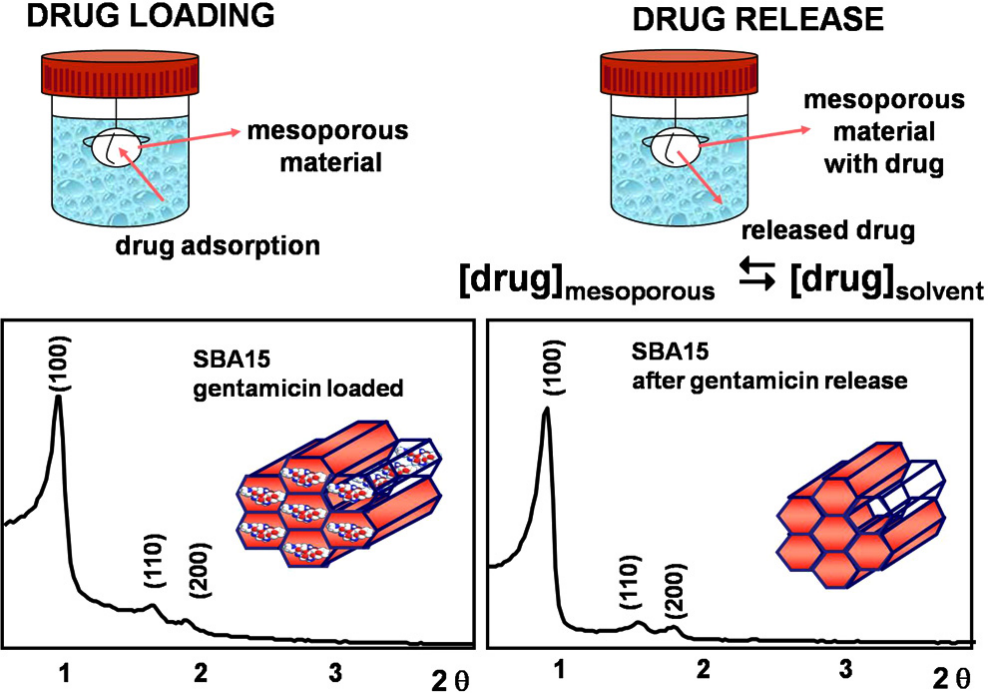
Scheme of the processes of drug loading and release. XRD patterns of SBA-15 ordered mesoporous materials loaded with gentamicin and after the drug release.

Essentially, the process of drug adsorption on the surface of mesopores is carried out by soaking the silica mesoporous solid in a solution containing the drug at certain conditions. In Fig. (**8**) can be observed that the adsorption of gentamicin, a well-known antibiotic, into the pores of SBA-15 does not modify the mesoporous hexagonal planar ordering [64]. On the other hand, the drug release tests are carried out by immersing the drug-loaded samples into a buffered solution that mimicks the conditions in the human plasma. Moreover, as it can be also seen in Figure 8, the mesoporous structure of SBA-15 does not undergo any modification after releasing the drug. This indicates that the mesoporous structures are stable for loading and releasing drugs.

This fact is also confirmed by the N_2_ adsorption-desorption experiments carried out in gentamicin-loaded SBA-15 and in the same materials after the releasing of drug molecules. As it shown in Fig. (**9**), the release of the drug from the pores of SBA-15 yields a surface area and pore volume very close to the value for as-synthesized SBA-15 (usually S_BET_ = 800 m^2^/g and V_P_ = 1.2 cm^3^/g). The pore diameter is also larger after the release of gentamicin since the adsorbed drug molecules are occupying part of the surface area of the mesopores, confirming that this is the mainly location for the loaded drug.

The release kinetics of the drug is usually studied as a function of time in order to determine the rate constants for desorption of drug molecules from the mesopores. It is worth mentioning that generally for mesoporous materials, the release profiles exhibit a pronounced initial burst release effect in the very initial testing time, followed by a very slow release pattern. The initial burst is attributed to the immediate dissolution and release of the portion of the drug located on and near the surface of the pore entrances. In addition, it is also commonly observed in release profiles that the complete desorption of the drug is seldom found. This fact is usually attributed to the equilibrium achieved at the end of the desorption process that favors the partial retention of the drug in the mesopores. This is more evident in the case of functionalized mesoporous matrices, where the interaction between the drug molecules and the modified silica walls is stronger [74].

Silanol groups are effectively present in the walls of silica mesoporous materials. This is a common feature with the above-described bioactive glasses. If the same bioactivity tests are performed in order to determine if an apatite layer can grow on the surface of silica-based mesoporous materials and there is a positive response, we would have another alternative material for bone regeneration, with the added value of confining inside the pores not only drugs, also peptides and other species that accelerate the bone regeneration. This is depicted in Fig. (**10**) and certainly, the results have been positive [16,76]. A layer built up of apatite nanocrystals grows on the surface of the mesoporous material, throughout the process, the mesoporous structure remains with few damages. Therefore, we are dealing with materials that exhibit the ability to regenerate bone, and the option of containing molecules to contribute in this process [62,77].

## 5. TEMPLATE GLASSES

In the preceding pages, it has been presented the state-of the-art in the research in silica materials for biomedical applications. The synthesis methods of mesoporous silica can be used for producing materials with the same compositions of bioactive glasses. In this way, the proper combination of both the excellent bioactive behavior of the glasses presented in Section 3 with the open texture and large surface areas of mesoporous materials will yield materials with potential applications in the biomedical fields.

These so-called template glasses are, in fact, derived from the use of micellar surfactants in the synthesis of equivalent compositions to those of bioactive glasses. The template glasses have to be synthesized employing a variation of the common preparation procedure of the mesoporous materials, for avoiding the precipitation of undesired phases during the synthesis. This modified procedure is known as the evaporation induced self-assembly (EISA) method [78], which is based in the formation of the micelle surfactant during the elimination of the solvent by evaporation. This method ensures the incorporation of Ca^2+^ cations in the SiO_2_ matrix, which are responsible for the enhancement of the bioactive behavior.

When comparing the results of bioactivity tests and surface area analysis of both conventional sol-gel glasses and template glasses, several facts can be observed. First, as it can be seen in the TEM image of Fig. (**11**), there is an ordered mesostructure and subsequently an opener texture than that observed for conventional sol-gel glasses. Moreover, the surface area of the template glasses is approximately the double of that found for equivalent sol-gel glasses, in terms of SiO_2_ content, as it can be seen in the N_2_ adsorption isotherms of Fig. (**12**). This is attributed to the presence of an ordered mesoporosity that would potentially lead to a better and more homogeneous adsorption of drugs and a higher contact area for the physiological fluids. This fact allows predicting that the kinetics of the apatite formation shall be much higher than that found for sol-gel glasses [18].

Indeed, the template glasses are more bioactive, in terms of the nucleation and growth of apatite on the surface of materials, than the conventional sol-gel glasses. The SEM images in Fig. (**12**) show that the surface of template glasses with large SiO_2_ content and a highly ordered mesostructure, is fully covered with an apatite layer within less than one single day of immersion in physiological conditions *in vitro*.

## 6. DISCUSSION

Along the present review, our aim was condensing the state-of-art in the research of silica materials with medical applications. Some of them are currently in clinical use like the bioglass, glass-ceramics, silica nanospheres and Fe_2_O_3_-doped silica nanospheres for magnetic targeting. Some other silica-based materials, like star gels, template glasses and ordered mesoporous materials are nowadays under testing and the initial assays do confirm that can be used for clinical applications.

Among all the medical applications that are described for these materials, we have pointed up two of them in this review. One of them was the use of these materials in the preparation of bioactive devices, since them show possibility to regenerate the bone tissue. In this classification, we included the bioglasses, glass ceramics, star gels, template glasses and ordered mesoporous materials. On the other hand, the silica-based materials can hold and release drugs and biologically active intermediates. In some cases, such molecules are encapsulated, as in the case of silica nanospheres, and in other by showing an ordered mesoporous ceramic matrix that allows hosting molecules for a controlled delivery in the adequate location in the physiological environment.

Summarizing, all this is shown in the scheme of Fig. (**13**). From all the reported data for silica materials useful for the medical practice, it can be deduced that the work in this area is just beginning. The possibilities are enormous, not only in the work that is in progress for processing these silica materials as pieces and devices with designed and hierarchical porosity, similar to that found in the bones of vertebrates. Such silica-based materials, therefore, could be excellent choices for designing substrates for tissue engineering.

## 7. CONCLUSIONS

Different silica-based materials can be employed for designing devices for biomedicine either for drug delivery systems or for bioactive materials in prosthetic applications. The current research is being focused in the development of devices that serve as clinical materials combining the above mentioned scopes. These silica-based systems are excellent candidates for designing substrates for biotechnology.

## Figures and Tables

**Fig. (1):**
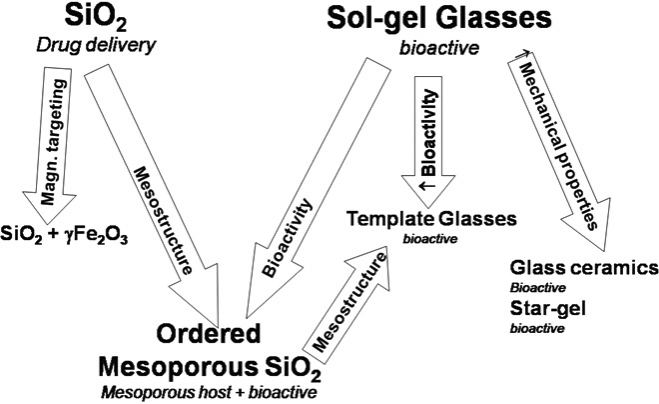
Scheme of the main families of silica-based materials used for biomedical applications.

**Fig. (3):**
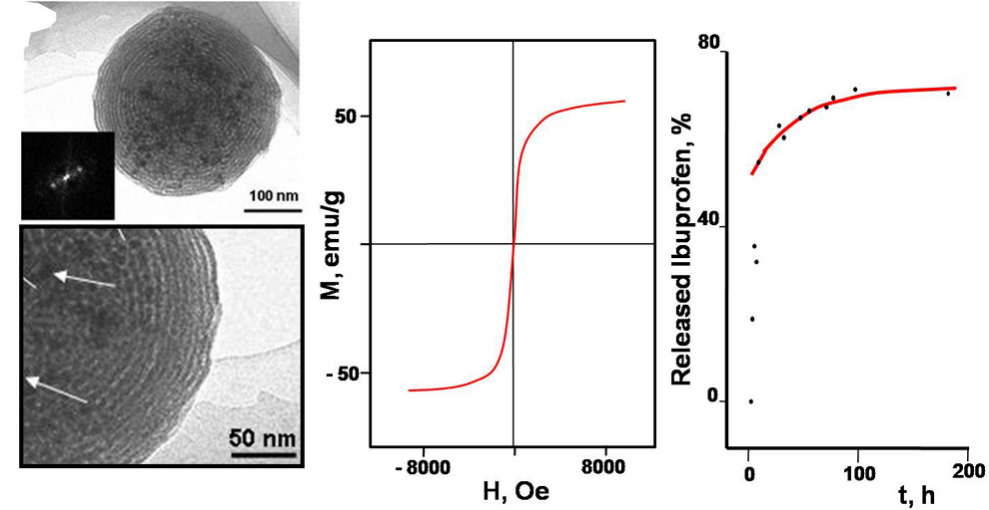
TEM images of SiO_2_·γ-Fe_2_O_3_ nanospheres obtained by pyrosol method. The arrows indicate the situation of the γ-Fe_2_O_3_ nanoparticles in the silica matrix. Magnetization curve and ibuprofen release profile are also shown.

**Fig. (4):**
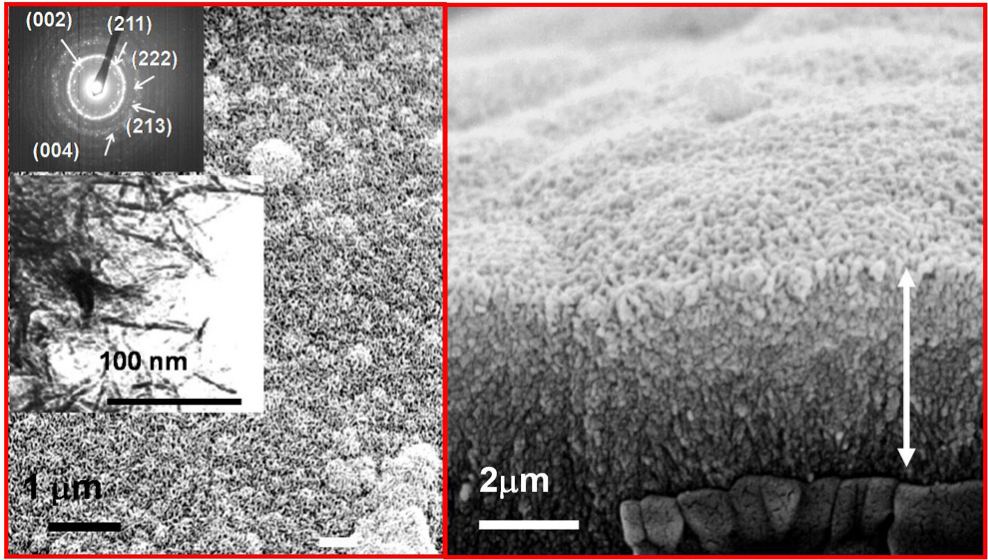
SEM and TEM images of the surface of _80_Si_17_Ca_3_P glass after 3 days of soaking in SBF at 37°C. Right side image shows the transversal cut of the material showing the aggregates of the apatite layer formed on the surface of the bioactive glass.

**Fig. (5):**
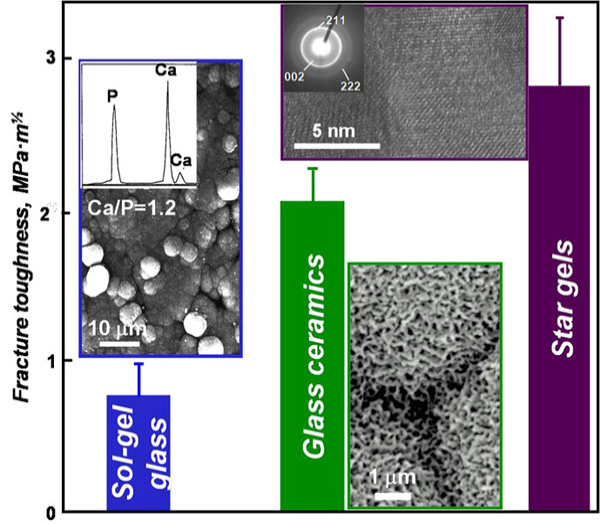
Comparison of the fracture toughness of sol-gel glass, glass ceramics and Star gels. For sol-gel glasses, the inset show the formation of spherical aggregates, which composition is closely related to that of the bone apatite. In the case of glass-ceramics, the formation of the aggregates of bonelike apatite layer is dependent of the soluble areas in the surface responsible for the bioactivity and the borders between aggregates are due to non-bioactive insoluble areas. For star-gels, the TEM image of the inset shows the amorphous nature of the materials with quasi-crystalline nuclei similar to the apatite and responsible for the bioactive behavior.

**Fig. (6):**
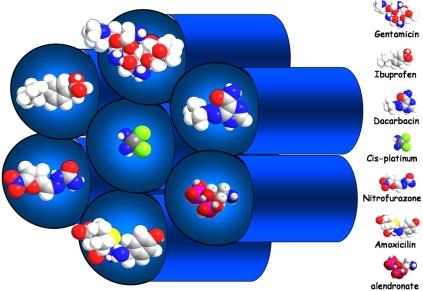
Schematic representation of the adsorption of drug molecules (ibuprofen, alendronate, erythromycin, gentamicin, vancomycin and cis-platin) into a hexagonally ordered mesoporous material.

**Fig. (7):**
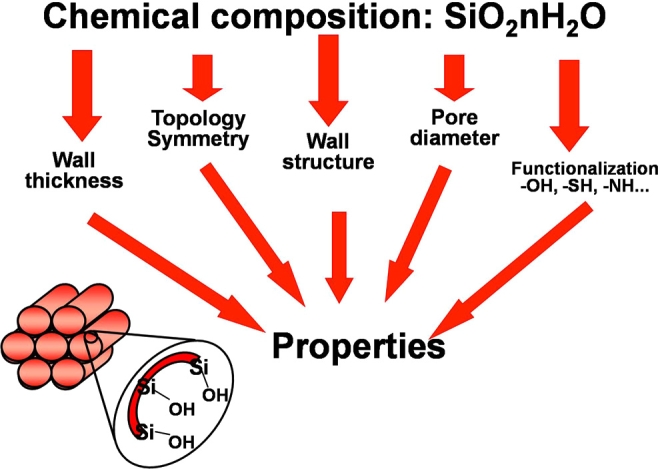
Scheme of the factors that affect to the properties of ordered mesoporous silica.

**Fig. (9):**
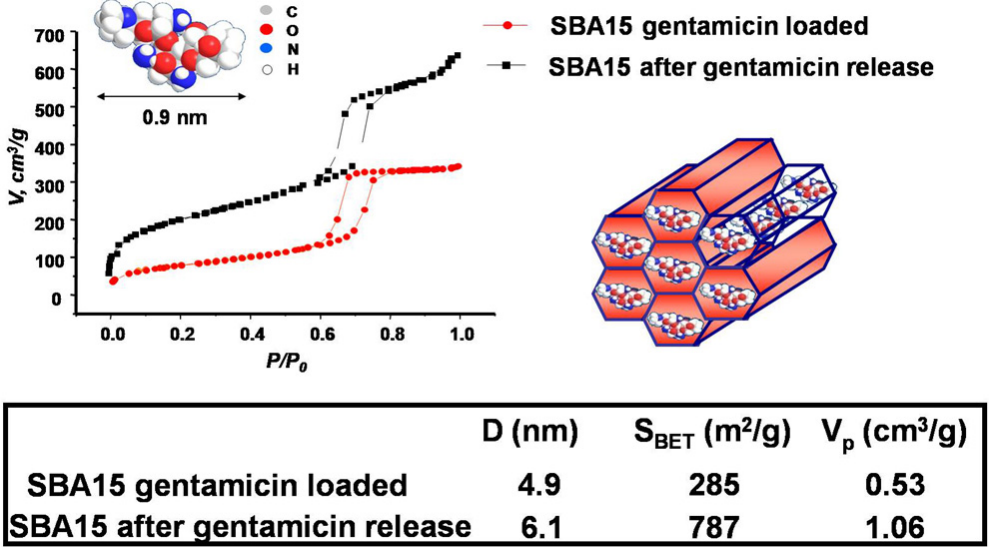
N_2_ adsorption isotherms and surface area values of SBA-15 ordered mesoporous materials loaded with gentamicin and after the drug release.

**Fig. (10):**
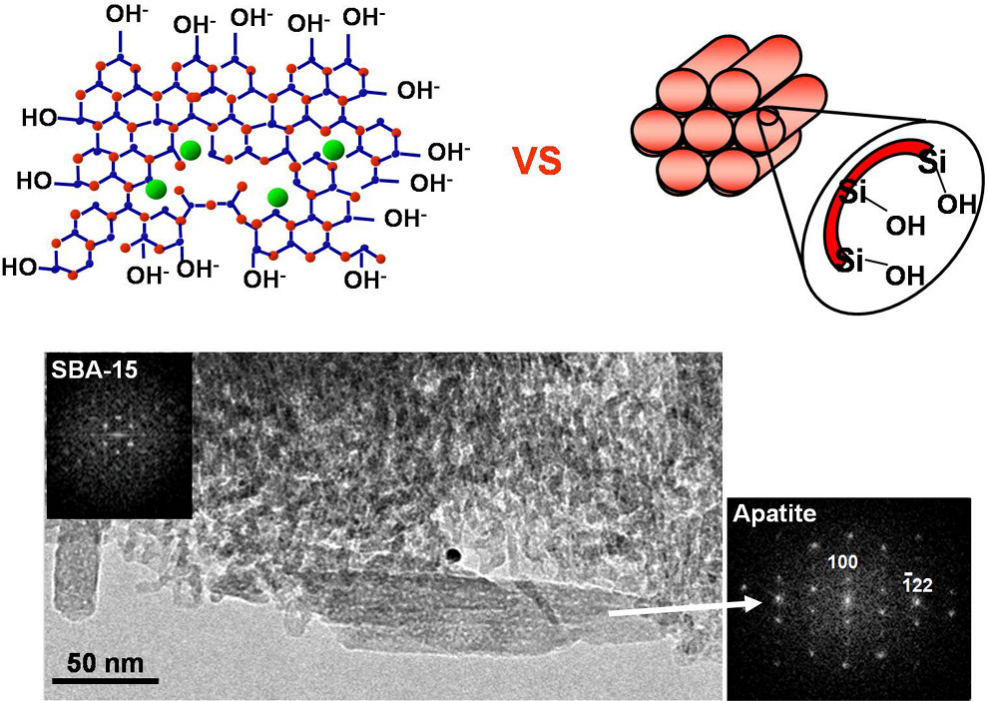
Scheme of the similarities, in terms of silanol (Si-OH) covering of the surface, between bioactive sol-gel glasses and ordered mesoporous materials. TEM image and ED pattern showing the formation of apatite on the surface of ordered mesoporous silica after soaking in SBF.

**Fig. (11):**
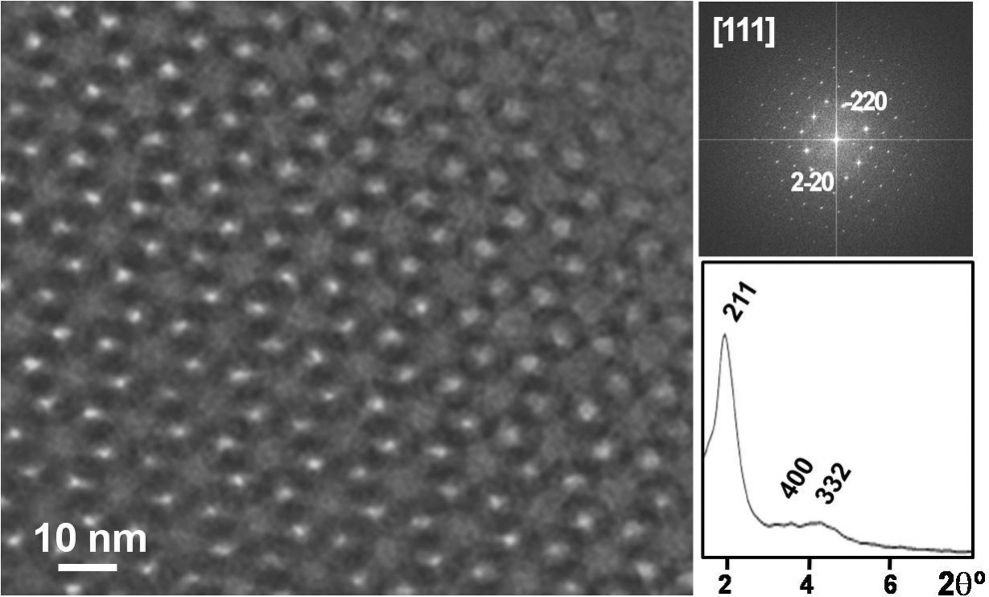
TEM image, Fourier-transform ED image and XRD pattern of one template glass with composition 85% SiO_2_, 10% CaO and 5% P_2_O_5_.

**Fig. (12):**
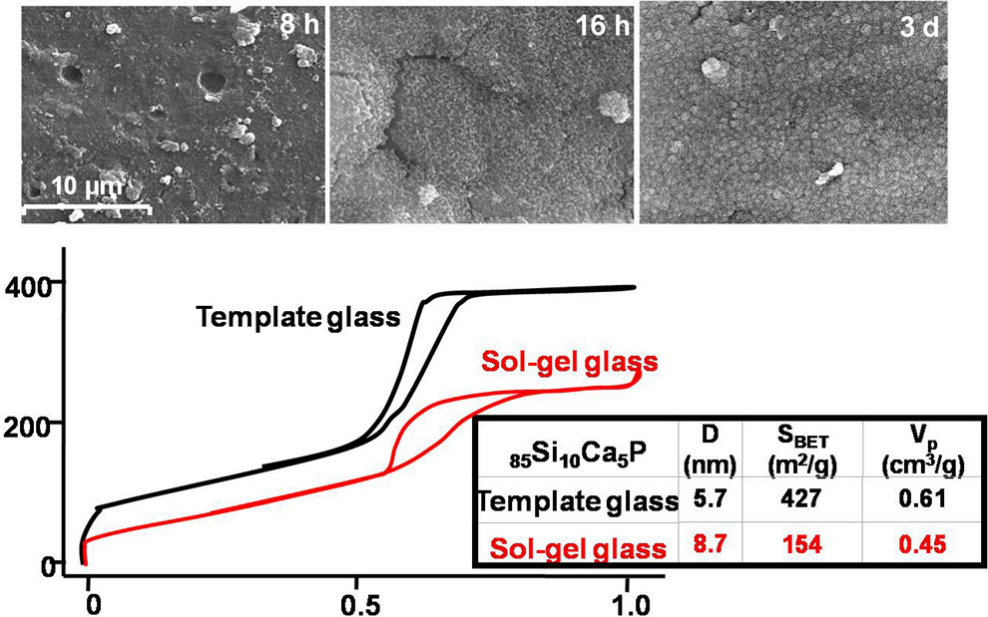
SEM images of one template glass with composition 85% SiO_2_, 10% CaO and 5% P_2_O_5_ after soaking in SBF for several periods. The comparison of the N_2_ adsorption isotherms of the template glass with a parent glass with similar composition is shown.

**Fig. (13):**
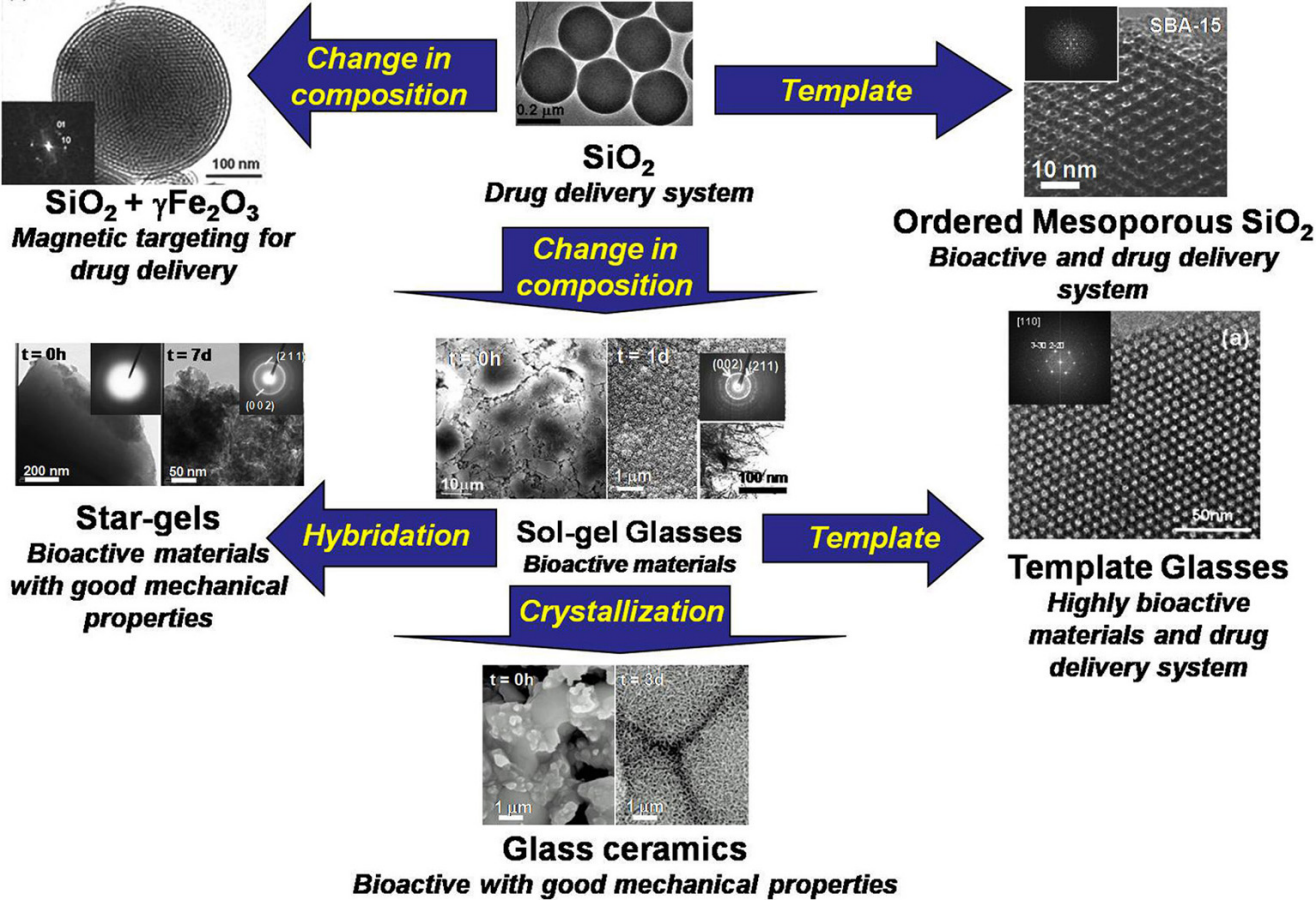
Scheme of the relationship between all the discussed families of silica-based materials with biomedical applications.
